# Clinical and histological evaluation of a dual sequential application of fractional 10,600 nm and 1570 nm lasers, compared to single applications in a porcine model

**DOI:** 10.1007/s10103-021-03460-5

**Published:** 2021-11-06

**Authors:** Igor Snast, Moshe Lapidoth, Assi Levi

**Affiliations:** 1 Laser Unit, Division of Dermatology, Rabin Medical Center, 39 Jabotinsky St, 4941492 Petah Tikva, Israel; 2 Sackler Faculty of Medicine, Tel Aviv University, 6997801 Tel Aviv, Israel

**Keywords:** CO_2_, Fractional, Histology, Laser, Porcine, 10,600 nm, 1570 nm, Sequential

## Abstract

The sequential application of fractional ablative/10,600 nm/CO_2_ followed by 1570 nm non-ablative laser treatment might produce better results than applying either laser treatment alone. However, histological data regarding the safety of this combination is lacking. This study aimed to assess and compare clinical effects, histological tissue damage, and wound healing after monochromatic and sequential fractional laser treatments. In this prospective porcine model study, three adult female pigs were each irradiated using three different wavelengths: (a) monochromatic fractional ablative CO_2_ laser; (b) monochromatic fractional non-ablative 1570 nm laser; (c) sequential fractional 10,600 nm/CO_2_ followed by 1570 nm laser treatment. There were six power levels in the monochromatic 1570 nm laser, five in the 10,600 nm/CO_2_, and five in the sequential treatment. The immediate skin reaction (ISR), crusting and adverse effects, was evaluated across different time points throughout the healing process. Wound biopsies were taken at immediately after (0) and at 3, 7, and 14 days after irradiation. Depth and width of craters, and width of coagulation zone were measured and compared. Similar ISR and crusting score values were obtained following the monochromatic and sequential irradiation in a similar dose–response manner. During 14 days of follow-up, the skin looked intact and non-infected with no signs of necrosis. The mean depth and width of craters were comparable only at the maximal energy level (240 mJ) of CO_2_ laser, with the coagulation size greater after the sequential treatment. In histology, a similar wound healing was evident. On day 3, crusts were observed above all lesions as was epithelial regeneration. The sequential irradiation with 10,600 nm/CO_2_ and 1570 nm lasers did not pose any additional risk compared to the risk of each laser alone.

## Introduction

In 2004, Manstein et al. introduced fractional photothermolysis (FP) [1]. This technique creates microscopic vertical channels in the skin surrounded by viable tissue. Consequently, the neighboring undamaged tissue allows for rapid healing of these damaged columns, and a shorter overall downtime for patients.

FP can be applied with a wide variety of lasers, both ablative and non-ablative. The ablative fractional CO_2_ laser has proven safe and effective not only for facial photoaged-related indications, but also for off-face [2]. It has also proven to be effective and safe for the treatment of pathologies in many other types of fields including otolaryngology, and plastic and general surgery [3, 4]. The non-ablative 1540–1570 nm laser permits deep penetration and selective targeting of water-containing tissue, creating a rise in dermal temperature and consequent collagen remodeling. Non-ablative laser skin resurfacing thus appears to be ideally suited for patients who are either unable or unwilling to undergo an ablative laser procedure, due to the associated prolonged downtime, or for those with only mild cutaneous pathology.

It was only recently that the combination of fractional non-ablative 1570 nm and ablative CO_2_ lasers for skin rejuvenation has been investigated in only few studies, demonstrating reductions in downtime and pain during treatments, and better results of fine wrinkles reduction than with either laser alone [5, 6]. However, these studies included a small number of subjects, and histological analysis of the skin ultrastructure and function was not performed.

The aims of the current study were to characterize tissue damage and wound healing caused by the monochromatic and sequential application of fractional non-ablative 1570 nm and ablative 10,600 nm/CO_2_ lasers, and to assess procedure-related adverse events by performing histological analysis in a porcine model.

## Materials and methods

### Animal model

The study was conducted after obtaining the approval of the institutional ethics committee (#31/2020). Three domestic female pigs with white skin and no obvious pigmentation were carefully selected. The animals were examined for possible disease or abnormal conditions, and included in the trial only after found suitable following clinical examination.

Crossbreed pigs were determined to be the most suitable model for this study due to the similarities between porcine and human skin with regard to thickness of the dermis and epidermis, density of dermal appendages, dermal collagen and elastic content, physical and molecular responses to various growth factors, and function of the immune system. Adult pigs were selected due to their skin thickness. The cutaneous tissue was the target tissue in this trial; thus, it was less desirable to have the subcutaneous tissue involved as would be expected in younger pigs with thinner cutaneous tissue. We chose to irradiate the belly since it is comparatively thin and accounts for approximately 18 percent of surface area of the entire animal. As depth and texture of the skin vary along the belly and sides of the pig, the location of the radiated area was carefully considered and grids were tattooed 5 days in advance to enable adequate recovery of the skin. Laser irradiation was administered only on white areas without any pigmentation/lesions in order to reduce adverse events. It was done relatively far from the tattooed spots that had been marked using a plastic template. Prior to irradiation, the animals were anesthetized with IM administration of Ketamine (20 mg/kg) + Xylazine (2 mg/Kg), intubated, and connected to mechanical ventilation equipment. Vital signs were monitored and recorded during the procedure.

### Laser treatment

The Alma Hybrid device (Alma Lasers Ltd., Caesarea, Israel) used for this study is a multi-technology platform that incorporates the CO_2_/10,600 nm tube laser (70/30 Watt) and 1570 nm fiber laser (15 W). The beam delivery system is a lightweight, spring-balanced, 7-joint, articulated arm. It includes two arms and a 3-knuckle end joint to which the surgical scanner applicator (ProScan) is attached. Three types of irradiation were investigated: 1570 nm, 10,600 nm, and sequential irradiation of the two wave lengths (WLs), where the 10,600 nm WL was first applied followed immediately by the 1570 nm. The settings are presented in Table 1. There was a single control, with four power levels in cases of monochromatic administration and four to five power levels (five for 10,600 nm WL and four for 1570 nm WL) for sequential administration, which were chosen following bench tests. An additional independent factor was pulse duration.

**Table 1:** Setting of the study

Sample #	Wavelength	CO_2_ power (W)	CO_2_ on time (ms)	1570 power (W)	1570 on time (ms)	Energy 10,600 nm (mJ)	Energy 1570 nm (mJ)
1	CO_2_	10	0.4			4	
2		20	1.8			36	
3		30	1.8			54	
4		30	4			120	
5		60	4			240	
6	1570			3	8		24
7				7	4		28
8				7	10		70
9				10	4		40
10				12	8		96
11				12	12		144
12	CO_2_ and then 1570	1	1.8	3	10	1.8	30
13		10	0.4	1	1	4	1
14		10	0.4	12	8	4	96
15		20	1.8	6	2	36	12
16		30	1.8	6	2	54	12
17		30	4	12	4	120	48
18		60	2	12	4	120	48
19		60	4	12	4	240	48
20	Control						

### Immediate skin reaction evaluation

**Fig. 1:**
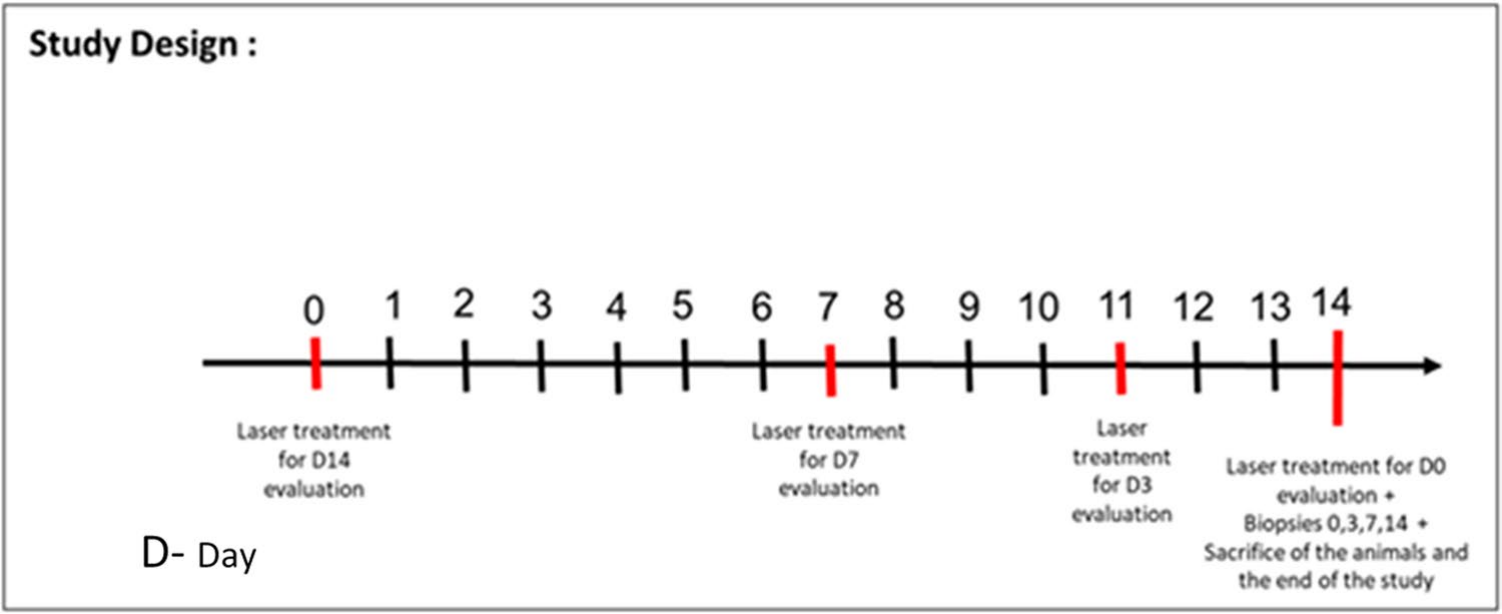
Trial timelines and study duration. Four time points were used: acute, 3, 7, and 14 days after radiation

Figure 1 presents the timeline of the study. The immediate skin reaction (ISR) was evaluated at days 1, 7, and 11 of the study, immediately after skin irradiation. As the standard deviation divided by square root of the sample size (SEM) for each participant did not differ compared to the combined SEM from all three pigs (*n* = 9), the combined SEM was used for comparing ISR between lasers. ISR score was determined according to the following scale: 0—no immediate skin reaction, 0.5—light erythema, 1—erythema, 1.5—light red wound, 2—red wound, and 2.5—dark red wound (rosewood color). In addition, all skin layers were thoroughly observed to assess parameters related to wound healing including epithelial regeneration, inflammation of the dermis, fibroblast proliferation, and presence of necrosis in the wounds.

### Crusting evaluation

Crusting was evaluated at 3, 7, and 14 days after irradiation. SEMs were calculated for each setting. Crusting level was assessed according to the following scale: 0 – normal, 0.5 – barely detected, 1 – mild, 1.5 – mild-to-moderate, and 2 – moderate.

On days 7 and 14, crusting was calculated relative to the 3-day scores.

### Histological evaluation

On days 0, 3, 7, and 14 after irradiation, 4 mm and 6 mm biopsies were acquired from all three pigs and H&E staining was performed on all of them. Time points were selected based on wound healing phases in mice, as established by Braiman-Wiksman and colleagues [11], and as those stages have been described in humans and found to be similar in pigs, with age variations.

Depth and width of craters, and width of coagulation zone were measured using a Nikon Eclipse E200 microscope DeltaPix SW, InSight 5.3.11. The crater dimension measurements were made by choosing at least three independent craters on each slide.

### Statistical analyses

A two-tailed *t*-test was used to compare dimensions between the pigs for each parameter. None was found to be significant; therefore, data were summarized into a pooled value comprising values of all pigs.

Analyses are narrative and descriptive, with results presented as SEMs. Student’s *t*-tests are 2-tailed, with a *P*-value of ≤ 0.05 considered statistically significant.

## Results

### Immediate skin reaction evaluation

**Fig. 2:**
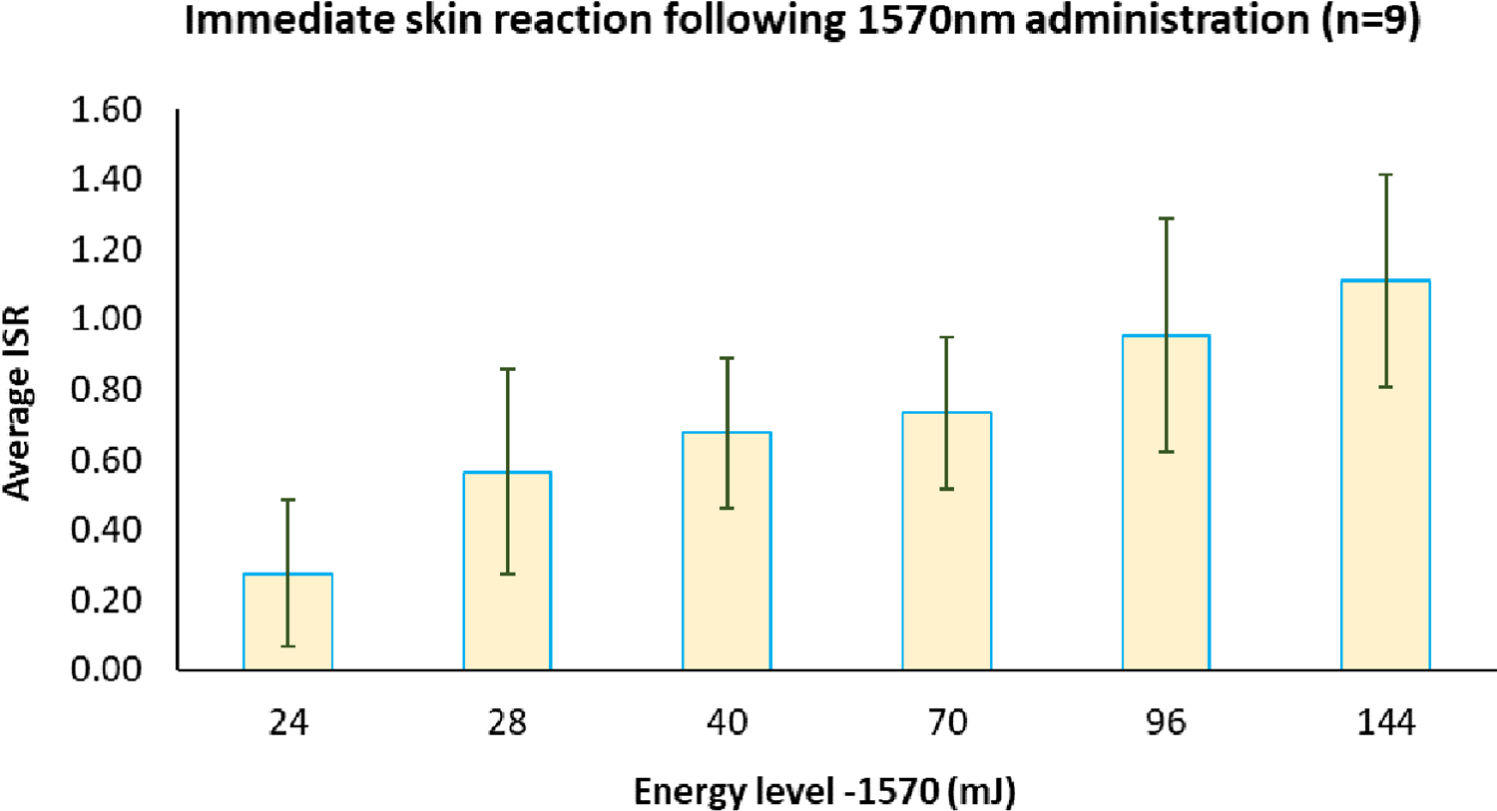
Immediate skin reaction following 1570 nm laser irradiation

**Fig. 3:**
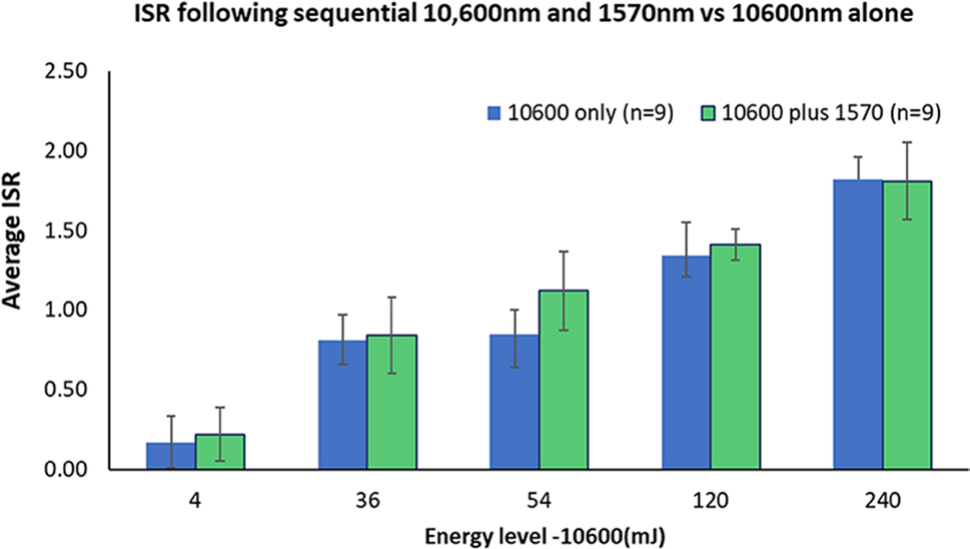
Immediate skin reaction following 10,600 nm laser irradiation as compared to sequential irradiation. The 1570 nm laser energy levels ranged between 1 and 96 mJ (Table 1)

Figure 2 shows ISR after 1570 nm irradiation, and Fig. 3 shows ISR after 10,600 nm irradiation, as compared with fractional treatments of 10,600 nm/CO_2_ followed by 1570 nm laser treatment. In all cases, skin reaction was found to be proportional to the energy level in a dose-dependent manner. As expected, 1570 nm irradiation was less aggressive as compared to 10,600 nm and to the sequential irradiation. ISR score values obtained following the monochromatic 10,600 nm and sequential irradiations showed a similar dose–response manner with no significant differences. At every visit during follow-up, the treated skin was carefully observed, and pigmented and textural modifications documented. The skin looked intact and non-infected with no necrosis, edema, or pus.

### Crusting level

**Fig. 4:**
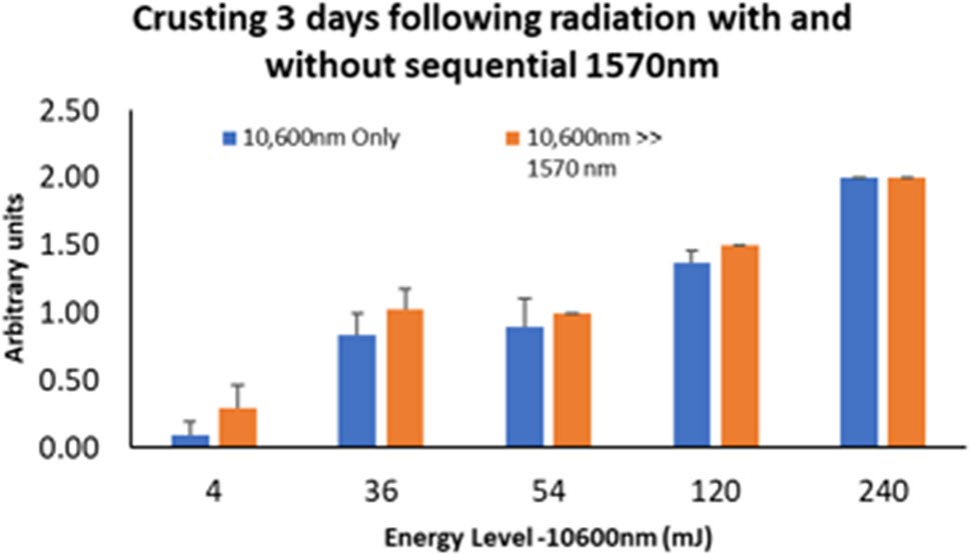
Crusting 3 days post 10,600 nm irradiation, as compared to sequential irradiation. The 1570 nm laser energy levels ranged between 1 and 96 mJ (Table 1)

**Fig. 5:**
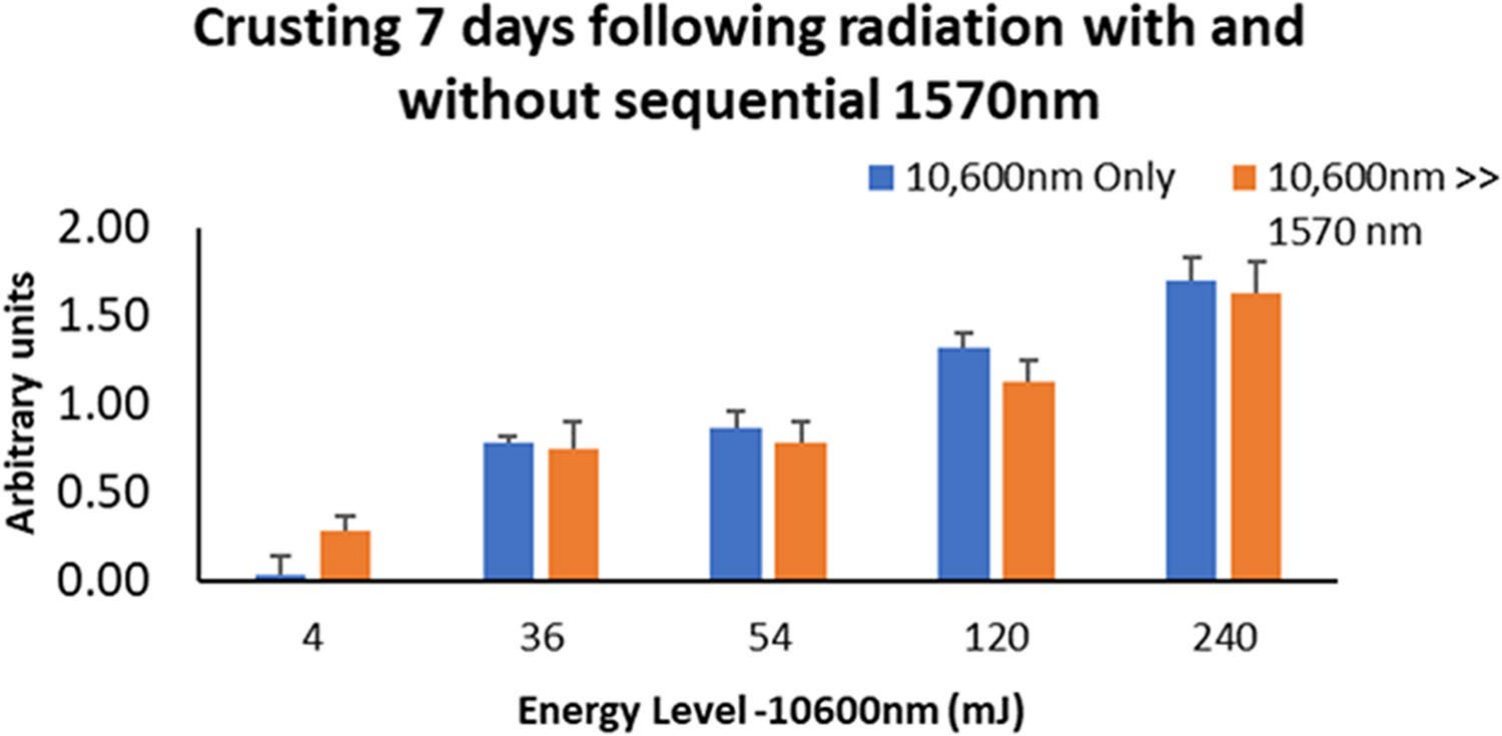
Crusting 7 days following 10,600 nm irradiation, as compared to sequential irradiation. The 1570 nm laser energy levels ranged between 1 and 96 mJ (Table 1)

Similar to the ISR results, crusting level was relatively higher at high-energy levels in a dose-dependent manner, and 1570 nm was found to be less aggressive compared to 10,600 nm. The scores were found to consistently decrease over time for all wavelengths and settings, in direct proportion to the energy level used. Crusting triggered by the 1570 nm technology was profoundly weaker as compared to ablative treatment. Similar crusting scores were found following 10,600 nm and sequential irradiation at 3, 7, and 14 days after irradiation (Figs. 4 and 5).

### Histological analysis

#### Measurement of crater’s depth, width, and coagulation zone

The non-ablative irradiation (1570 nm) formed V-shape craters of necrotic tissue. Maximal depth of necrotic tissue was found to be 980 µM ± 49.2 µM, obtained at 144 mJ. Maximal width of coagulation was 344.4 µM ± 12.3 µM. Depths and widths of coagulation were directly proportional to energy levels.

**Fig. 6:**
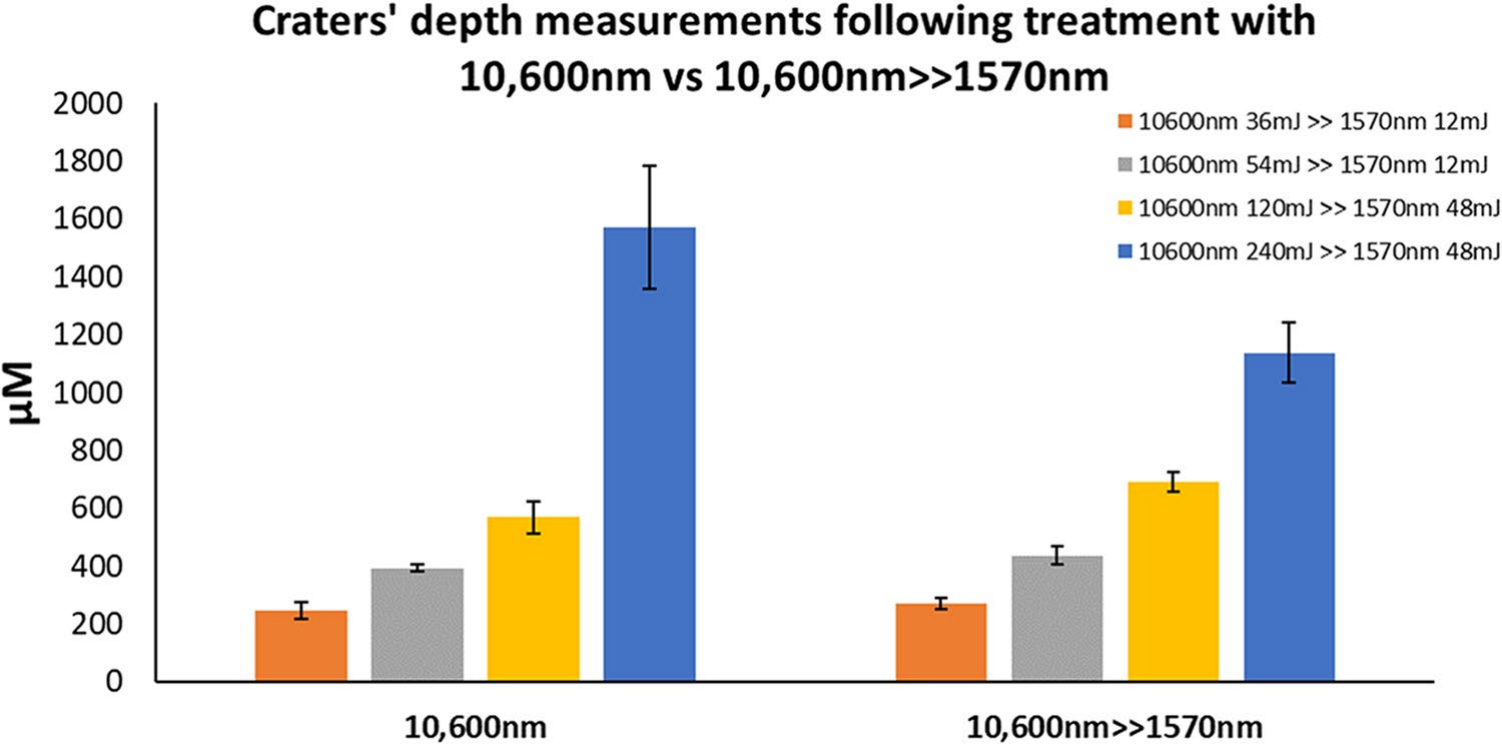
Crater depths following 10,600 nm irradiation as compared with sequential irradiation

**Fig. 7:**
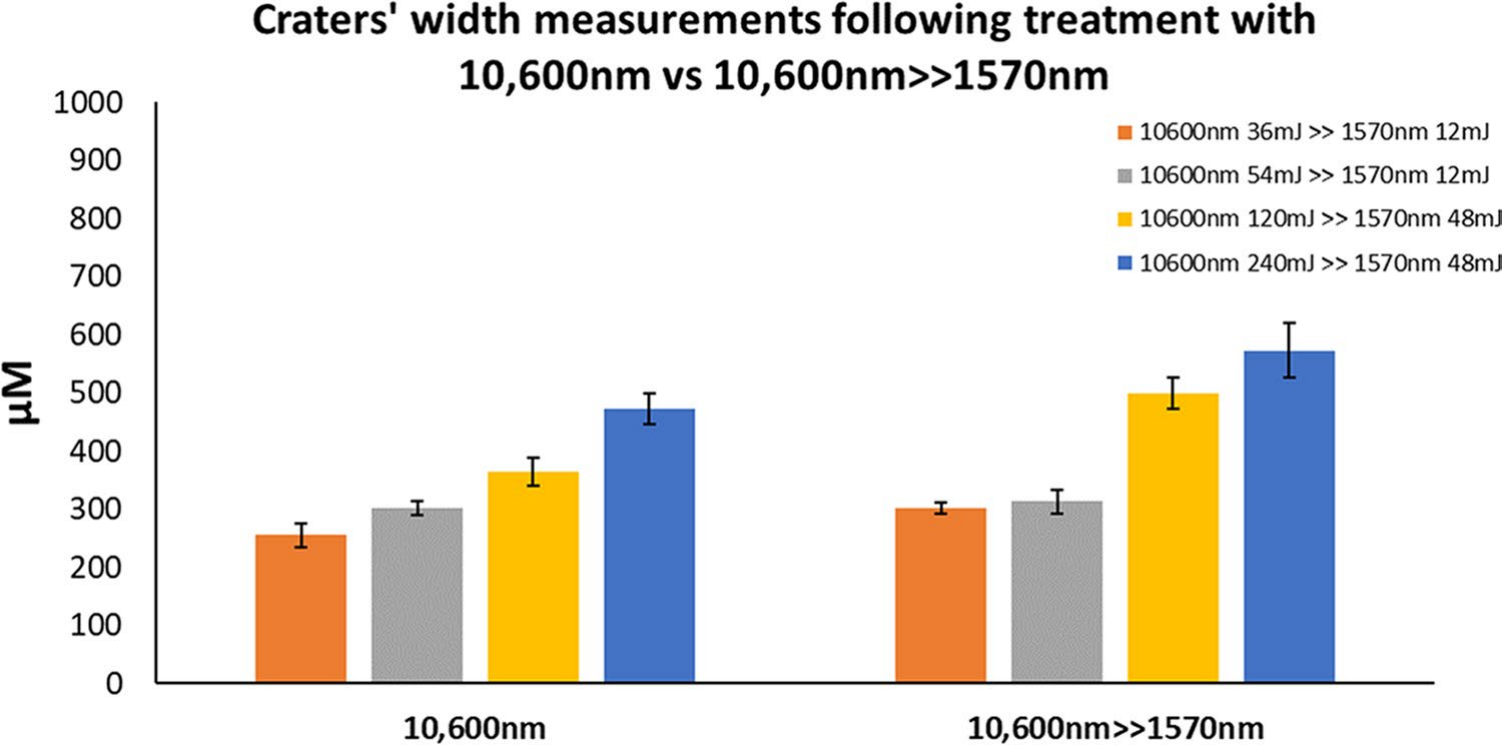
Crater widths following 10,600 nm irradiation as compared with sequential irradiation

**Fig. 8:**
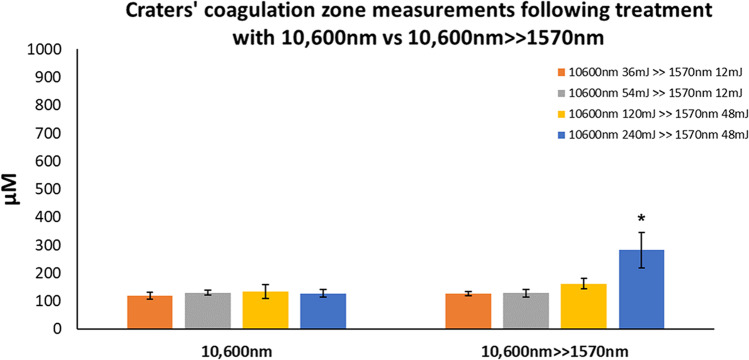
Craters’ coagulation zone following 10,600 nm irradiation as compared with sequential irradiation

Mean depth, width, and coagulation ± SEM were used to compare the ablative alone to sequential irradiation (Figs. 6, 7, and 8). All three parameters increased in direct proportion to energy level at both settings, with maximal mean depth and width observed at maximal energy level (240 mJ) for 10,600 nm WL (depth—1573 µM ± 212 µM, width—473 µM ± 27 µM) and sequential irradiation (depth—1138 µM ± 102 µM, width—573 µM ± 47 µM). These differences were not statistically significant. Coagulation size following 10,600 nm irradiation ranged between 119 µM ± 11 µM and 128 µM ± 13 µM, and was found to be steady. The only significant difference was obtained at 240 mJ following sequential irradiation as compared to irradiation with the 10,600 nm WL alone (282 µM ± 64 µM and 128 µM ± 13 µM respectively, *P* < 0.01).

### Histology following irradiation

#### 1570 nm

**Fig. 9:**
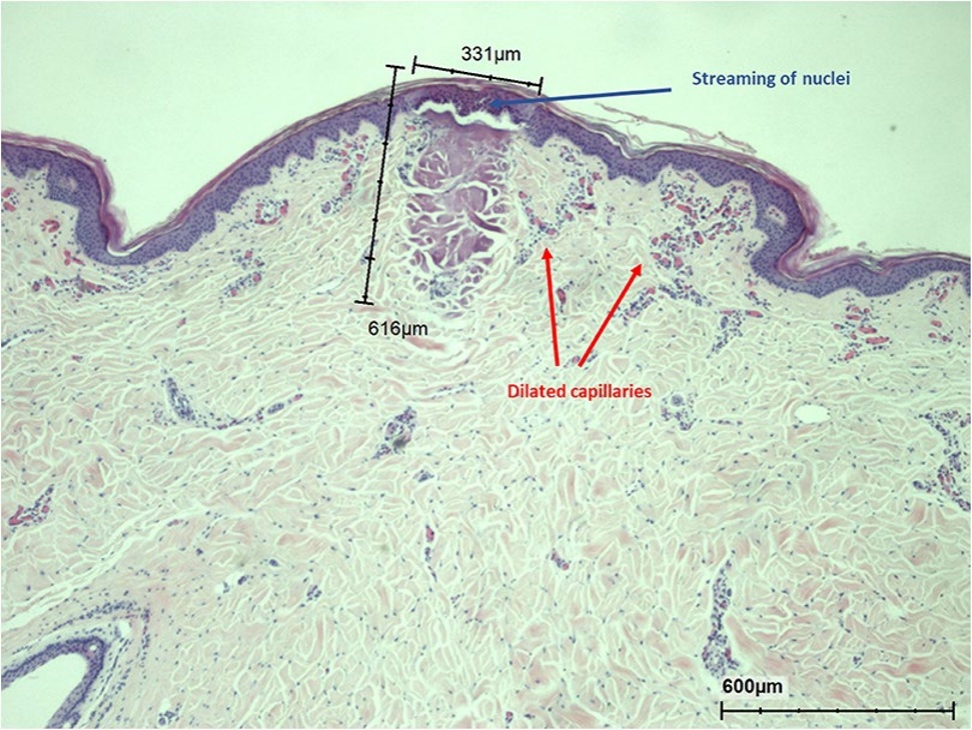
Histology of acute effect following administration of 40 mJ 1570 nm wavelength

Acute effects were observed following application of 1570 nm laser (40 mJ), showing the foci of thermal necrosis with no ablation (Fig. 9). Streaming of nuclei in the epidermis was found, with condensation of collagen and pyknosis of cells of capillary walls in the dermis. The surrounding skin appeared within normal ranges. There was, however, mild dilation and congestion of superficial dermal capillaries without inflammation, hemorrhage, or edema. At 3 days after irradiation, large remnants of thermal necrosis were observed in foci with increased leukocyte infiltration. At 7 and 14 days after irradiation, epidermal regeneration was complete with minimal or no inflammation. Focal thermal necrosis was observed above the normal epidermis.

#### nm vs the sequential irradiation

**Fig. 10:**
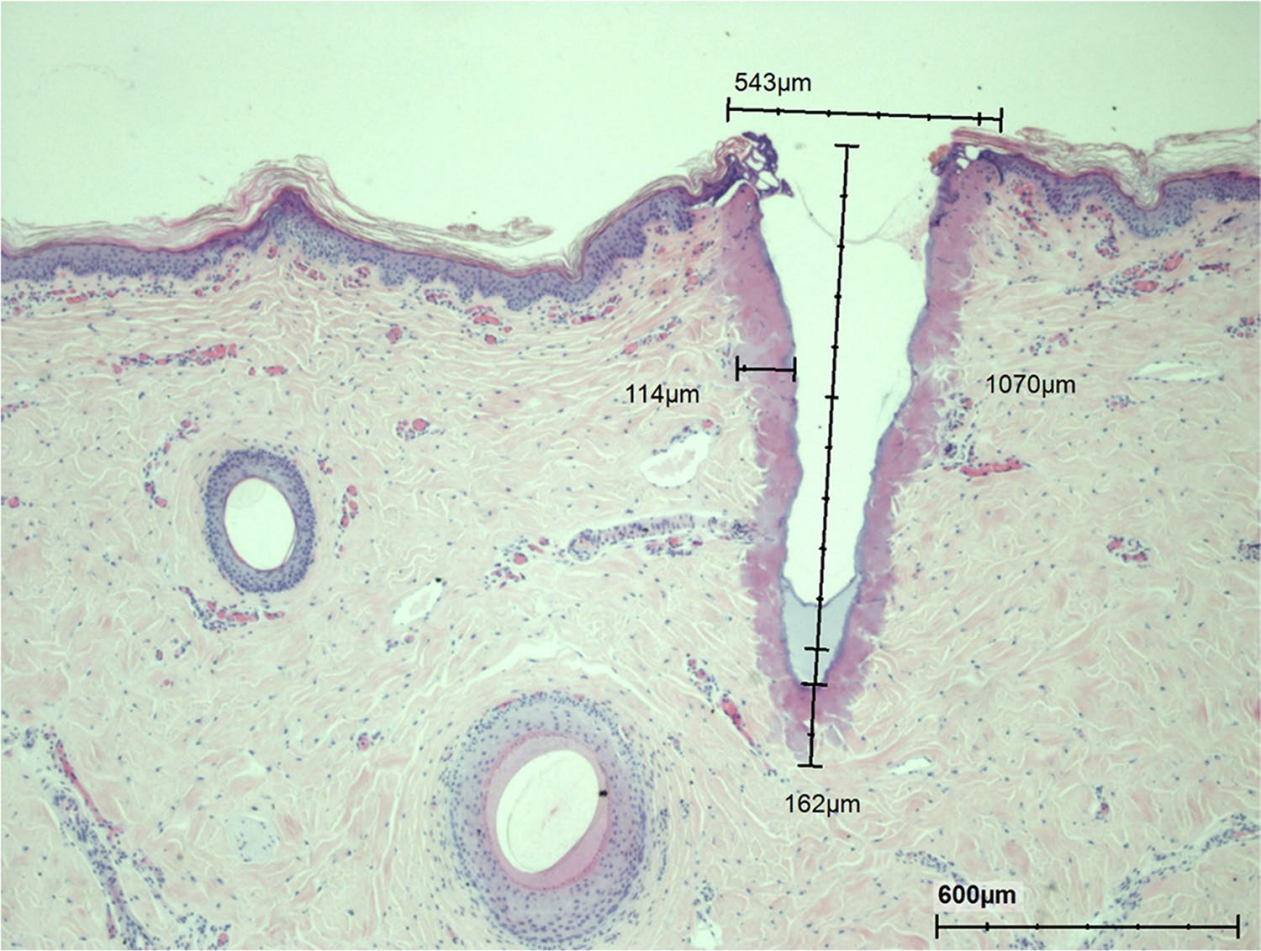
Histology of acute effect following administration of 240 mJ 10,600 nm wavelength

**Fig. 11:**
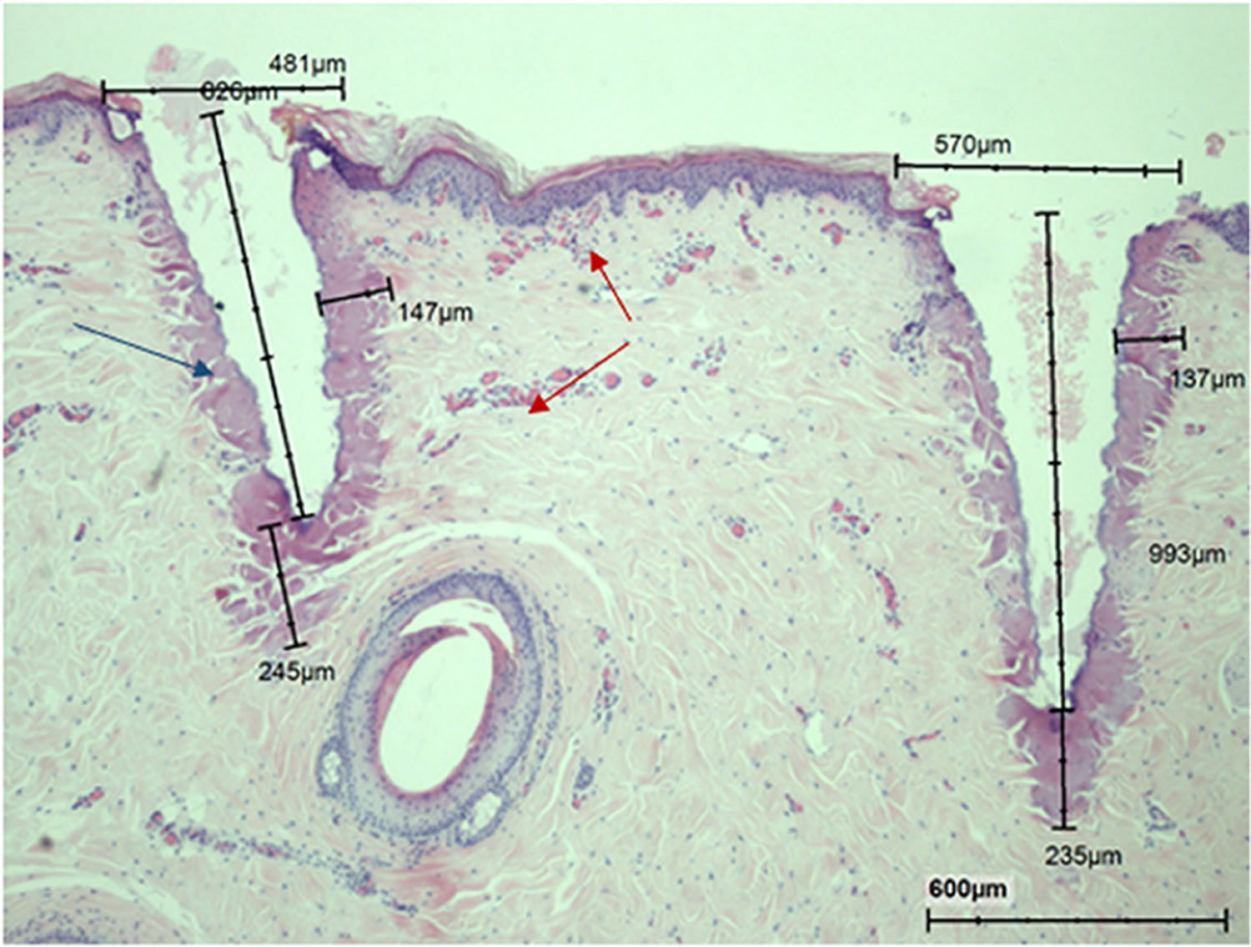
Histology of acute effect following sequential radiation of 240 mJ 10,600 followed by 48 mJ 1570 nm. Note: Similar shape of craters and blood congestion adjacent to the craters (red arrows). The narrow necrotic rim surrounds the crater (blue arrow)

When comparing ablative to sequential irradiation, craters were observed following 10,600 nm WL (240 mJ) irradiation as compared to sequential irradiation with 10,600 nm (240 mJ) and 1570 nm WL (48 mJ) (Figs. 10 and 11). Similar V-shaped foci of ablation with a uniform peripheral rim of thermal necrosis were observed. The surrounding skin looked normal. There was, however, mild dilation and congestion of superficial dermal capillaries. No inflammation, hemorrhage, or edema was found. Capillaries close to the narrow necrotic rim were dilated.

**Fig. 12:**
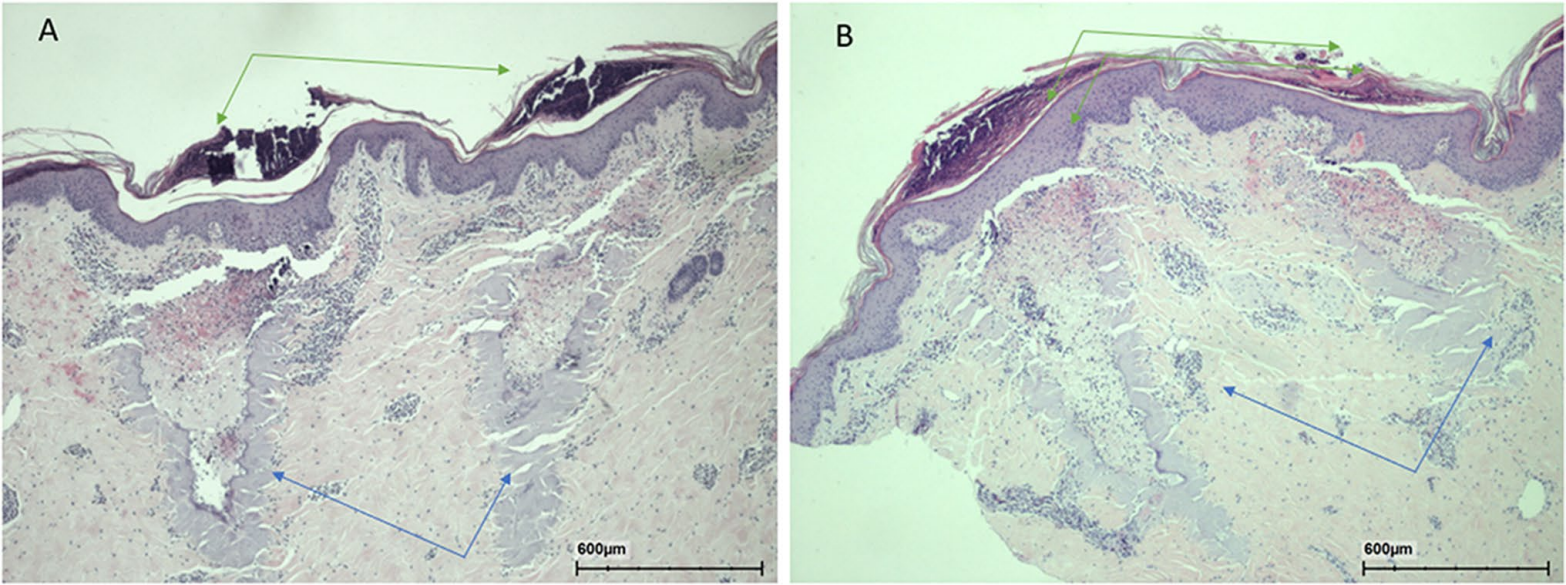
Histology at 3 days following 240 mJ of 10,600 nm (**A**) and sequential radiation (**B**). Necrotic areas of the craters (blue arrows) and crusts above them (green arrows) are shown. Infiltration of leukocytes was observed within the craters

With both modalities at 3 days after irradiation, small or large remnants of thermal necrosis were observed in foci (Fig. 12). Mild granulocyte infiltration was detected in the area. Crusts were seen above foci.

**Fig. 13:**
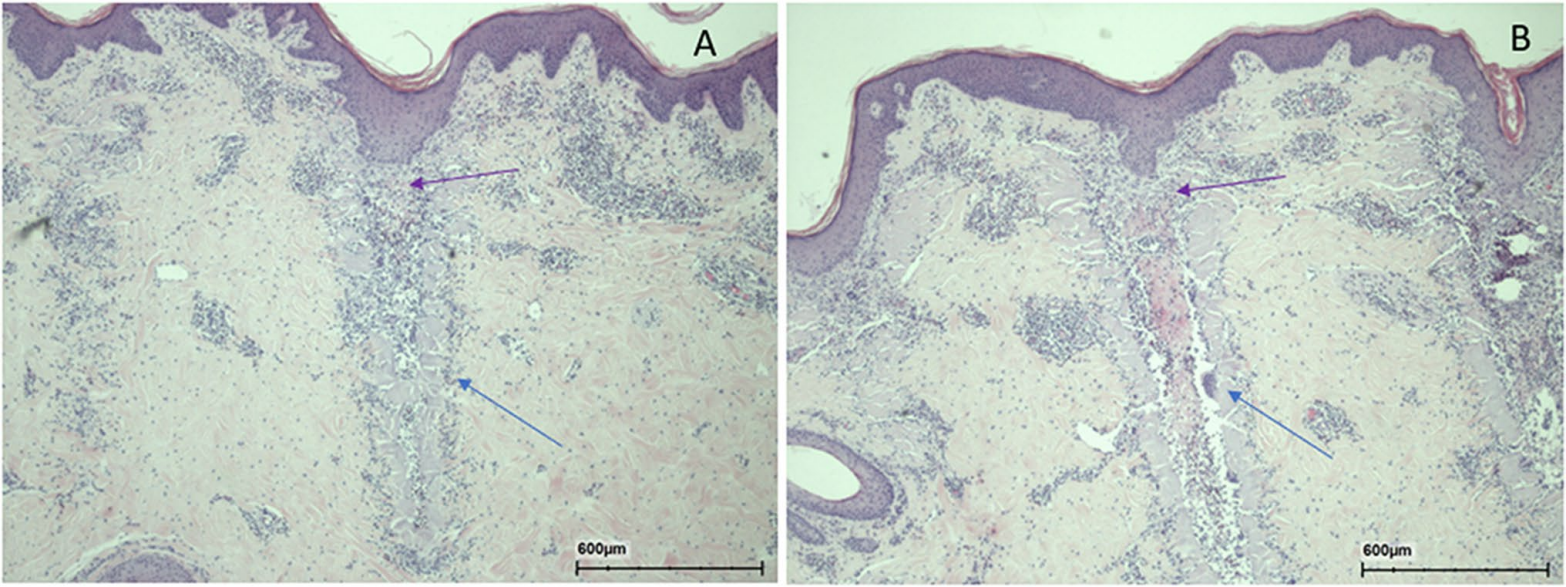
Histology at 7 days following 240 mJ of 10,600 nm (**A**) and sequential radiation (**B**). Necrotic areas of the craters are shown with the blue arrows. Crusts are not seen and most probably have been detached. Infiltration of leukocytes and also early fibroblast proliferation (purple arrows) was observed in the area

Seven days after irradiation, foci of variable sizes were observed (Fig. 13), and in the large foci, central fibrin was detected surrounding thermal necrosis. Mild leukocyte infiltration with fibroblast proliferation was also detected, and epidermal regeneration found to be complete.

**Fig. 14:**
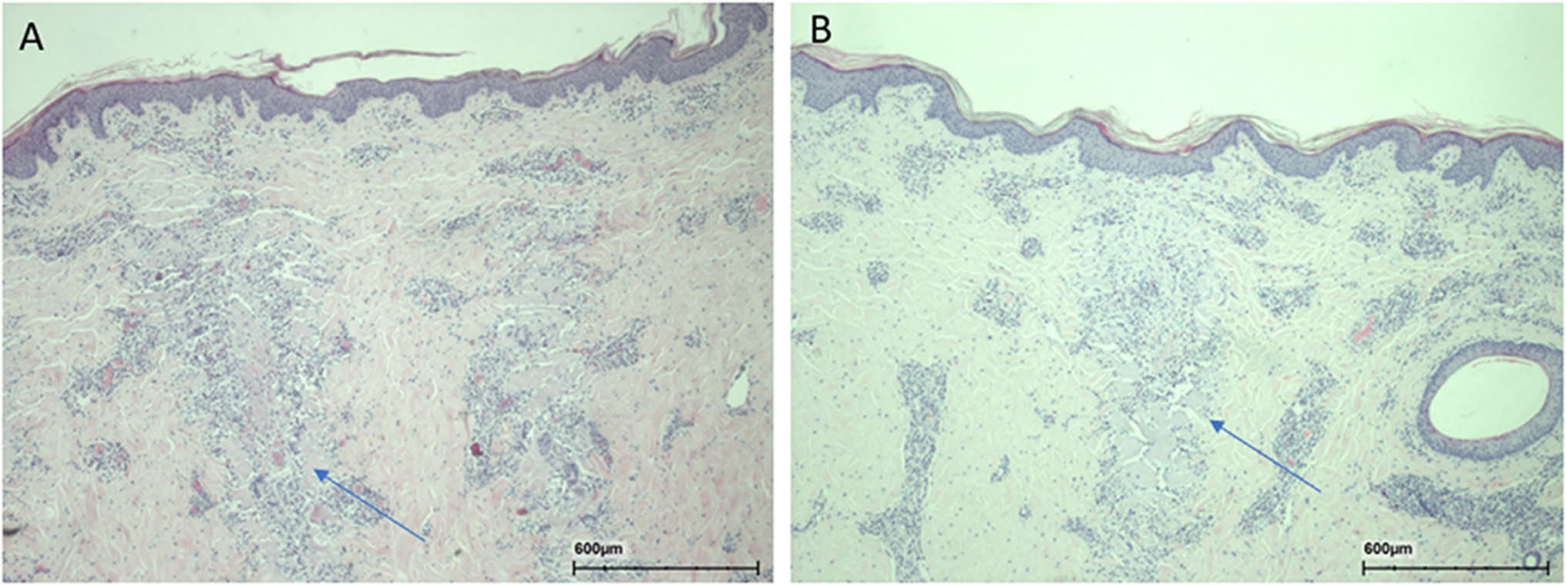
Histology at 14 days following 240 mJ of 10,600 nm (**A**) and sequential radiation (**B**). Remnant of necrotic areas of the craters is shown with the blue arrows. Infiltration of leukocytes was observed in the area and fibrin deposition

Fourteen days after irradiation, no crusts were found (Fig. 14). There were remnants of foci in various sizes. Multinucleated giant cells and proliferating fibroblasts were seen near lesions.

## Discussion

In this study, tissue damage and wound healing were assessed following the use of 10,600 nm, 1570 nm, and their sequential administration. Similar reaction patterns (i.e., depth, width, and coagulation zone of the craters) were observed when either 10,600 nm or sequential emission of 10,600 nm and 1570 nm was administered. Also, the wound healing process followed a similar course.

Non-ablative fractional lasers are associated with a low rate of adverse effects and may be applicable for almost every patient, but repeated treatments might be required to achieve the desired result [7]. In contrast, ablative fractional lasers have faster results but are associated with higher rate of complications, and longer healing time and downtime. The combination of ablative and non-ablative lasers may offer a better outcome than using each independently. Accordingly, Mezzana et al.’s study of 20 patients showed that the simultaneous combination of fractional CO_2_ (15 W power with a pulse duration of 0.5 ms) and 1540 nm lasers compared to C0_2_ laser alone (20 W power with a pulse duration of 1 ms) reduced the downtime and produced better results on fine wrinkles reduction [5]. Similarly, in another study of 20 patients, Kim and Cho [8] demonstrated that the combination of low-energy fractional CO_2_ laser resurfacing (pulse energy of 15 to 35 mJ, pulse duration of 0.03 ms) and non-ablative 1064 nm laser resurfacing yielded better results on acne scars compared to high energy fractional CO_2_ laser alone (pulse energy of 50 to 70 mJ, pulse duration of 0.03 ms), and reduced the downtime [8]. However, in those studies, histological analysis was not performed. The main strength of the current histopathological study was that we were able to assess and characterize tissue damage and wound healing caused by the monochromatic and sequential application of fractional non-ablative 1570 nm and ablative CO_2_ lasers. Indeed, we have shown that crater form and size, and thus the wound healing process, are similar in both treatment applications, supporting the safety of the sequential treatment. Additionally, none of the pigs developed any unexpected complication although aggressive settings were used. Importantly, the non-ablative technology did not modify the ablation of CO_2_ laser, but did increase the coagulation zone, which might be the reason for the improved efficacy of the combined treatment in clinical studies.

The main reason for using a porcine model in this safety study was the similarity between porcine and human skin [9, 10]. Three pigs enabled wide working surfaces with repeats at various energy levels, and adequate number of controls taken from different locations in the abdomen of the animals.

In histological analysis, the wounds were found to be limited to the dermis. Re-epithelialization was observed even at the highest energy settings. This might have been achieved via increases in keratinocyte proliferation. To the most, histological observations found evidence of necrosis, with or without leukocyte infiltration and sometimes accompanied with fibrin deposition, similar to the described features associated with ablative and non-ablative technologies. According to the histology, wound area was found to involve only the dermis and yielded crusts of various sizes. Crusts were found above foci even after 1570 nm irradiation, which is considered to be less aggressive as compared to the ablative laser. Similar to this study, visible crusts have been reported following non-ablative laser treatments in clinical trials [12].

No complications were observed in any pig. Erythema and edema are major side effects associated with both ablative and non-ablative lasers [13]. Additionally, ablative lasers may cause scarring, discoloration, and skin infections [14]. However, no complications were detected in the three pigs, and only crusting was documented following irradiation, highlighting the safety of the sequential treatment.

The main limitation of this study was the lack of efficacy measurements, i.e., collagen remodeling was not investigated. According to previous studies, following 1540 nm laser treatment the proportion of collagen bundles in the papillary dermis significantly increased 12 weeks after the last treatment [15]. Also, the elastic fiber framework in the papillary dermis significantly increased and new elastic fibers were seen in the upper dermis and the mid-dermis, while the previously disordered and fragmented elastic fibers normalized. Likewise, the 10,600 nm laser has been proven to induce collagen remodeling in multiple immunohistochemical studies performed [16]. This raises the important question whether the sequential treatment has a synergistic effect. Further histological studies are warranted in the future in order to address this important topic.

## Conclusion

According to this clinical and histological investigation, the combined irradiation of 10,600 nm and 1570 nm does not pose any additional risk compared to using each independently, and both options seem to be safe.
